# Phosphorylation of p130Cas initiates Rac activation and membrane ruffling

**DOI:** 10.1186/1471-2121-9-50

**Published:** 2008-09-15

**Authors:** Alok Sharma, Bruce J Mayer

**Affiliations:** 1 Department of Pharmaceutical Sciences, Massachusetts College of Pharmacy and Health Sciences, 1260 Elm Street, Manchester, NH 03101, USA; 2 Raymond and Beverly Sackler Laboratory of Genetics and Molecular Medicine, Department of Genetics and Developmental Biology, University of Connecticut Health Center, 263 Farmington Avenue, Farmington, CT 06030-3301, USA

## Abstract

**Background:**

Non-receptor tyrosine kinases (NTKs) regulate physiological processes such as cell migration, differentiation, proliferation, and survival by interacting with and phosphorylating a large number of substrates simultaneously. This makes it difficult to attribute a particular biological effect to the phosphorylation of a particular substrate. We developed the Functional Interaction Trap (FIT) method to phosphorylate specifically a single substrate of choice in living cells, thereby allowing the biological effect(s) of that phosphorylation to be assessed. In this study we have used FIT to investigate the effects of specific phosphorylation of p130Cas, a protein implicated in cell migration. We have also used this approach to address a controversy regarding whether it is Src family kinases or focal adhesion kinase (FAK) that phosphorylates p130Cas in the trimolecular Src-FAK-p130Cas complex.

**Results:**

We show here that SYF cells (mouse fibroblasts lacking the NTKs Src, Yes and Fyn) exhibit a low level of basal tyrosine phosphorylation at focal adhesions. FIT-mediated tyrosine phosphorylation of NTK substrates p130Cas, paxillin and FAK and cortactin was observed at focal adhesions, while FIT-mediated phosphorylation of cortactin was also seen at the cell periphery. Phosphorylation of p130Cas in SYF cells led to activation of Rac1 and increased membrane ruffling and lamellipodium formation, events associated with cell migration. We also found that the kinase activity of Src and not FAK is essential for phosphorylation of p130Cas when the three proteins exist as a complex in focal adhesions.

**Conclusion:**

These results demonstrate that tyrosine phosphorylation of p130Cas is sufficient for its localization to focal adhesions and for activation of downstream signaling events associated with cell migration. FIT provides a valuable tool to evaluate the contribution of individual components of the response to signals with multiple outputs, such as activation of NTKs.

## Background

Cell migration is a fundamental cellular process that is essential for embryonic development and many normal functions, such as wound healing and immunity. It also plays a role in various disease processes including tumor angiogenesis and metastasis [1,2]. Although our current understanding of cell migration is incomplete, research in the past 15 years has shed light on this complex process (see [2-7] for reviews and references).

Signaling cascades mediated by NTKs are believed to play a central role in cell migration. Knock-out mutation in mice of NTKs such as Src/Yes/Fyn [8], FAK [9] or Abl/Arg [10], or NTK substrates such as p130Cas [11] or paxillin [12], results in embryonic lethality, which has been specifically attributed to the failure of cell migration. Integrin receptor activation following adhesion to extracellular matrix is believed to be the primary stimulus to activate the signaling cascades mediated by NTKs. Following integrin receptor activation, autophosphorylation of FAK at tyrosine 397 [13-16] recruits Src [17] and p130Cas [18] leading to the activation of two separate pathways working in conjunction with each other. The first pathway involves activation of Erk via Grb2/SOS/Ras-MAPK pathway as a result of FAK phosphorylation at tyrosine 925 by Src [19,20] (but see [21]). Erk activates myosin light chain kinase (MLCK), which phosphorylates myosin light chain thereby promoting its interaction with actin, resulting in the generation of force required for cell movement [22-24]. MLCK activation also allows retrograde flow of actin in the lamellipodia [25], which brings about "periodic lamellipodial retractions" that bring activated signaling molecules from the front to the back, where they act ([26], see also [27]). The second pathway involves extensive phosphorylation of p130Cas [16,21] that promotes Crk binding [28], which in turn recruits DOCK 180 [24], leading to the activation of the Rho family GTPase Rac1 [24,28-30]. Rac1 activation promotes membrane ruffling, lamellipodium formation and actin reorganization [31] by acting on the WASP/WAVE family of Arp2/3 complex activators to stimulate actin polymerization [5,32,33].

In summary, it is now accepted that activation of Rac1 promotes membrane ruffling, lamellipodial extensions and actin reorganization, while Erk activation promotes the interaction of actin and myosin to generate the force for cell movement. Together, these actions generate cell polarity, where the coordinated movement of actin and myosin result in the formation of a leading edge and a trailing edge necessary for cell migration (see [1-7] for reviews).

NTKs such as Src are known to interact physically with a large number of substrate proteins such as enzymes, cytoskeletal proteins and adaptor molecules. Binding is mediated by the Src homology 2 (SH2) and SH3 domains of the kinases with phosphorylated tyrosine residues and proline-rich sequences on the substrate. The Src-substrate interaction induces the efficient phosphorylation of substrates, which in turn serves to initiate downstream signaling for cellular processes such as cell migration, differentiation, proliferation and survival [34-36]. However, due to the complexity of the network of protein interactions, and the fact that many proteins are phosphorylated simultaneously upon NTK activation, it has been difficult to determine which particular kinase-substrate interactions play a critical role in cell migration (or other effects).

p130Cas is one of the adaptor molecules that is known to be a substrate of NTKs such as Src. It was first isolated as a heavily tyrosine phosphorylated protein from v-Src- [37] and v-Crk-transformed cells [38-40], and it has been shown to play a role in cell transformation [41]. p130Cas is also thought to play an important role in cell adhesion, migration, growth factor stimulation, cytokine receptor engagement and bacterial infection (see [11] for references). However, determining which of these specific biological outputs occur as a direct result of the phosphorylation of p130Cas has been difficult. Moreover, there is an ongoing controversy over the mechanism of phosphorylation of p130Cas. The substrate domain of p130Cas is known to be tyrosine phosphorylated by Src directly after interaction of the RPLPSPP motif of p130Cas with the SH3 domain of Src [42]. However because p130Cas exists at focal adhesions in a macromolecular complex with FAK and Src [43], both of which possess tyrosine kinase activity, the relative role of FAK and Src in phosphorylating p130Cas is difficult to assess (see [44,45]).

At least four different models have been postulated for the phosphorylation of p130Cas in this complex [44]. Using FAK mutants in in vitro kinase assays and antibody labeling, Hanks and co-workers [44,46] have provided evidence for a model in which FAK autophosphorylation at Y397 results in recruitment of Src via its SH2 domain, while interaction of the SH3 domain of p130Cas with proline rich regions in FAK recruits p130Cas to FAK; Src then phosphorylates p130Cas bound to FAK. A similar conclusion has also been reached using Src mutations [45]. Support for the idea that FAK kinase activity is dispensable for phosphorylation of p130Cas was also provided by studies utilizing overexpression of FAK mutants in CHO cells [16,21], and the observation that phosphorylation of p130Cas is reduced in Src-/- cells [47,48].

**Figure 1:**
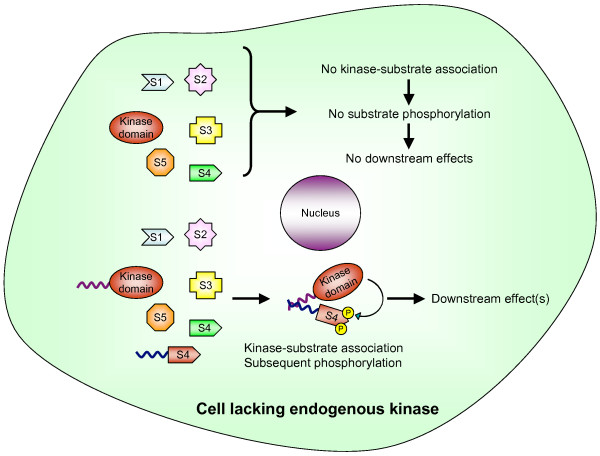
Schematic representation of the FIT approach. Removal of the substrate binding domains (SH2 and SH3) of NTKs (denoted as kinase domain) abolishes the association with and tyrosine phosphorylation of substrates (denoted as S1–S5). Transfection of FIT-compatible kinase and a FIT-compatible substrate (possessing artificial binding interface; represented by wavy line) promotes specific association between the kinase and substrate leading to its tyrosine phosphorylation (denoted by P) and hence downstream effect(s).

In order to determine which particular effects are mediated upon specific phosphorylation of p130Cas and to address the relative roles of Src and FAK in phosphorylation of p130Cas, we have utilized a methodology called the Functional Interaction Trap (FIT) [49-51]. Briefly, FIT utilizes an artificial binding interface composed of two leucine zipper coiled-coiled domains (ZipA and ZipB) [50-52]. One coiled coil segment is present in a constitutively active NTK lacking its SH2 and SH3 substrate binding domains, and the second in a substrate of interest. Expression of the modified kinase and substrate in a cell induces a specific interaction between them, resulting in the specific phosphorylation of only that substrate. Any biological output observed can thus be directly attributed to the phosphorylation of that particular substrate (Figure 1; see also [51]).

We have already shown that FIT can indeed promote a physical and a physiological interaction between two proteins of interest that can lead to a biological output by mimicking endogenous signaling pathways [50]. Our initial observation that FIT-induced phosphorylation of p130Cas induced in NIH 3T3 cells a bipolar shape with long, thin terminal processes [50] suggested that specific phosphorylation of p130Cas might be sufficient to induce actin cytoskeletal rearrangements. In this study, we have utilized FIT to study the specific biological output(s) mediated by the specific phosphorylation of p130Cas in more detail. Additionally, we have used FIT to address the controversy regarding the mechanism of p130Cas phosphorylation in focal adhesions.

## Results

### SYF and c-Src reconstituted cells as a biological system for FIT

To use FIT to determine the normal functional (physiological) consequences of tyrosine phosphorylation of specific substrates by NTKs, a cellular system is needed that lacks the NTK and as a result exhibits a defect in some aspect of cellular functioning. In such a system, if expression of FIT-compatible kinase and substrate can rescue the phenotype, this would provide strong evidence that phosphorylation of that particular substrate is essential for the biological output under investigation. SYF cells are mouse embryonic fibroblasts (MEFs) that lack the three Src family kinases detectably expressed in fibroblastic cells (Src, Yes and Fyn). These cells exhibit defects in cell migration [8], and therefore could serve as an excellent system to determine the biological consequences that result from FIT-mediated tyrosine phosphorylation.

**Figure 2:**
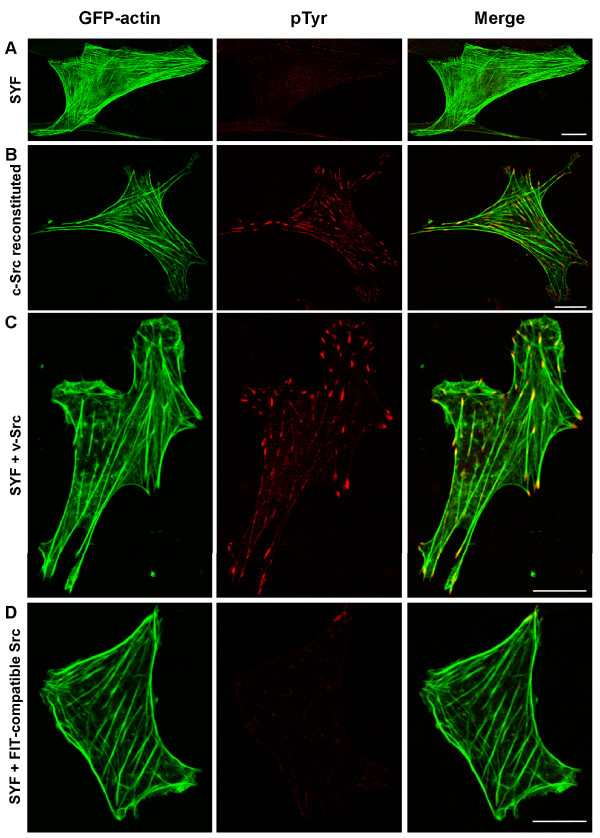
Tyrosine phosphorylation in SYF cells is increased by expressing c-Src or v-Src, but not by FIT-compatible Src. SYF cells were transfected with plasmids expressing GFP-actin alone (A) or in combination with v-Src (C) or FIT-compatible Src (D) while c-Src reconstituted cells were transfected with GFP-actin vector only (B). GFP-actin and anti-pTyr immunofluorescence were observed by confocal microscopy of fixed, permeabilized cells. Merged image is presented on right. Scale bars represent 10 μM.

We first tested the feasibility of using the SYF cells as a cellular system to study FIT-mediated responses. As reported previously [8], we found that SYF cells and SYF cells reconstituted with c-Src show no obvious differences in the organization of cytoskeleton and focal adhesions (Figure 2 and data not shown). However, as expected, the basal level of tyrosine phosphorylation was significantly different. Few, if any sites of tyrosine phosphorylation were observed in SYF cells by immunofluorescence (Figure 2A), which is in agreement with previous findings of reduced phosphotyrosine content of FAK that can be immunoprecipitated from SYF cells [8] and failure to detect significant tyrosine phosphorylation in SYF cells [53]. As expected, the c-Src reconstituted cells showed significantly more tyrosine phosphorylation, primarily at discrete punctuate sites at the ends of actin stress fibers, consistent with focal adhesions (Figure 2B). Transfection of v-Src in SYF cells also led to extensive tyrosine phosphorylation at focal adhesions (Figure 2C). In contrast, transfection of v-Src lacking the substrate binding SH2 and SH3 domains, but containing a coiled-coiled domain (ZipA) and an intact membrane targeting sequence (hereafter referred to as FIT-compatible Src), induced no significant increase in tyrosine phosphorylation in SYF cells (Figure 2D). These results demonstrate that SYF cells expressing FIT-compatible Src have sufficiently low background to study the biological effects of FIT-mediated phosphorylation of specific substrates.

### FIT-mediated specific phosphorylation of Src substrates increases tyrosine phosphorylation in SYF cells

Focal adhesions are macromolecular signaling complexes that link the extracellular matrix to the intracellular actin cytoskeleton [54,55]. The assembly and disassembly (turnover) of focal adhesion components is important for regulating cell adhesion and cell migration [9,16,54-57]. Many proteins believed to play a role in cell migration are localized at focal adhesions, including FAK, Src, Csk, NTK substrates such as p130Cas, paxillin and cortactin, MAPK, Ras, Rho and Rac, and protein tyrosine phosphatases (see [58,59], for references).

**Figure 3:**
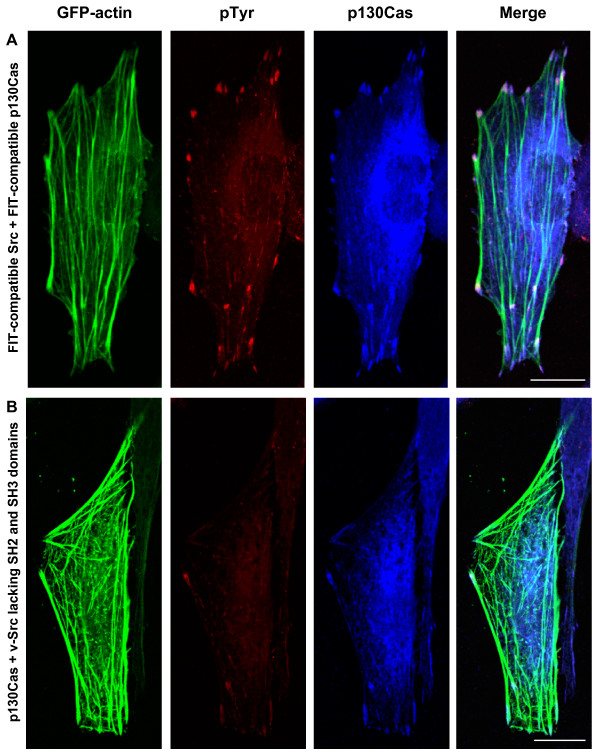
FIT-mediated specific phosphorylation of p130Cas in SYF cells leads to increased tyrosine phosphorylation of p130Cas at focal adhesions. SYF cells were transfected either with vectors expressing GFP-actin, FIT-compatible Src and FIT-compatible p130Cas (A) or with GFP-actin, ΔSrc (lacking substrate binding domains) and p130Cas, both lacking the artificial binding interface. Tyrosine phosphorylation was visualized by anti-pTyr and rhodamine while p130Cas localization was visualized by anti-Cas and Alexa-647. Merged image is presented on right. Scale bars represent 10 μM.

In order to observe directly whether the FIT-mediated phosphorylation of p130Cas promotes localization to focal adhesions, we expressed FIT-compatible Src and FIT-compatible p130Cas (p130Cas fused to ZipB, the coiled-coil segment complementary to ZipA) in SYF cells. Co-transfection of FIT-compatible Src and p130Cas resulted in increased tyrosine phosphorylation at focal adhesion sites (Figure 3A). Transfection of p130Cas alone (data not shown) or p130Cas and v-Src lacking SH2 and SH3 domains, both lacking the artificial binding interface (Figure 3B), do not lead to increased tyrosine phosphorylation at focal adhesions, indicating the specificity of this process. Moreover, tyrosine phosphorylation of p130Cas is apparently sufficient to recruit p130Cas to focal adhesions, since much more p130Cas is found at focal adhesions when it is tyrosine phosphorylated by FIT (Figure 3A) as opposed to when it is not (Figure 3B).

**Figure 4:**
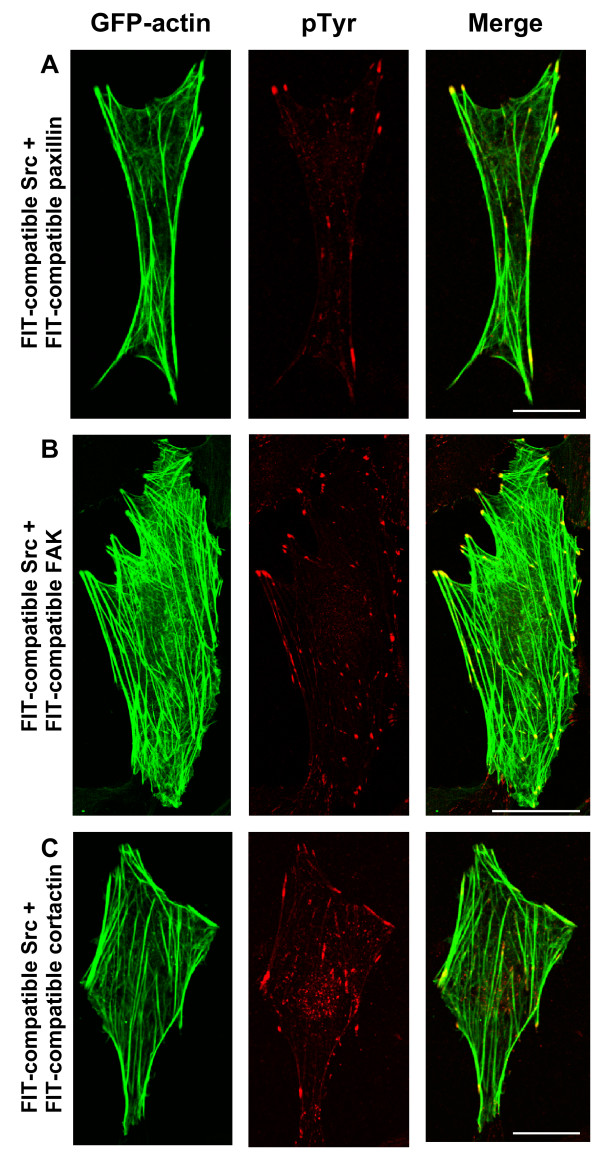
FIT-mediated specific phosphorylation of paxillin, cortactin and FAK in SYF cells. SYF cells were transfected with GFP-actin and FIT-compatible Src with FIT-compatible paxillin (A), FIT-compatible FAK (B) or FIT-compatible cortactin (C). GFP-actin and tyrosine phosphorylation were visualized as in figures 2 and 3. Merged image is presented on right. Scale bars represent 10 μM.

We also used FIT to induce the specific tyrosine phosphorylation of several other substrates of Src in SYF cells. FIT-mediated specific tyrosine phosphorylation of paxillin (Figure 4A) and FAK (Figure 4B) also increased the tyrosine phosphorylation at focal adhesions, while that of cortactin showed increased tyrosine phosphorylation at the cortical membrane in addition to focal adhesions (Figure 4C). The specific phosphorylation of p130Cas (or other substrates) by FIT could also be detected in NIH 3T3 or SYF cells by immunoprecipitation and western blot studies (data not shown, but see [50]). These results confirm that specific phosphorylation of substrates such as p130Cas by FIT occurs at discrete and specific sites within the cell, consistent with the normal subcellular distribution of the substrate.

### FIT-mediated specific phosphorylation of p130Cas results in activation of Rac1

**Figure 5:**
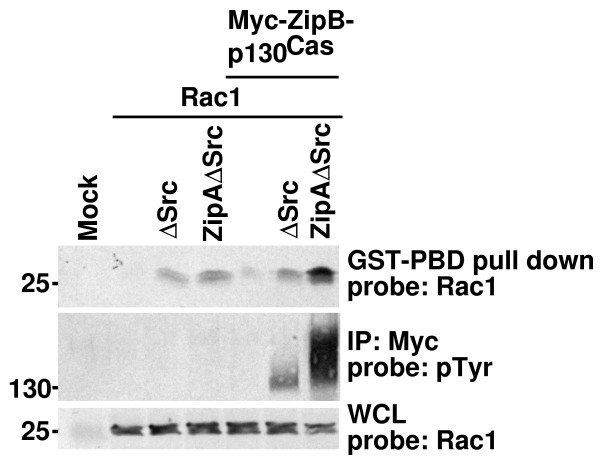
FIT-mediated specific phosphorylation of p130Cas leads to activation of Rac1. 293T cells were transfected with vectors expressing Rac1 and other proteins as indicated. Cell lysates were subjected to GST-PBD pull-down assay (top panel) or immunoprecipitation (IP; middle panel) and analyzed by western blot. Membranes were probed with anti-Rac1 (top panel) or anti-pTyr (middle panel). Whole cell lysates (WCL) were also probed by anti-Rac1 to determine the total amount of Rac1 expressed (bottom panel). FIT-mediated phosphorylation of p130Cas and subsequent activation of Rac1 is predicted to occur only in cells co-expressing FIT-compatible Src (ZipA-ΔSrc) and Myc-tagged FIT-compatible p130Cas (Myc-ZipB-p130Cas). ΔSrc = v-Src lacking SH2 and SH3 domains. Approximate position of molecular weight markers (kDa) is shown on left.

Signals that activate cell migration are known to activate Rac1, and phosphorylation of p130Cas is thought to activate downstream signals that activate Rac1 [29,30]. Since FIT relies on the assumption that appropriate downstream signaling cascades can be activated as a result of phosphorylation of a single substrate, we tested whether the FIT-mediated phosphorylation of p130Cas led to the activation of Rac1. We used a Rac1 pull-down assay as described previously [60] to test whether FIT-mediated p130Cas phosphorylation increased overall levels of GTP-bound (activated) Rac1 in cell lysates. As shown in figure 5, the level of activated Rac1 was much higher in cells expressing FIT-compatible Src and p130Cas than in controls, confirming that tyrosine phosphorylation of p130Cas (in the absence of significant phosphorylation of other substrates) is sufficient to lead to Rac1 activation.

### FIT-mediated specific phosphorylation of p130Cas results in increased membrane ruffling and lamellipodium formation

Activation of Rac1 is known to stimulate actin polymerization and membrane ruffling and is required for the protrusion of lamellipodia [29-31,46], and dominant negative Rac1 inhibits lamellipodium formation, membrane ruffling and cell migration [29,61,62]. In order to determine whether the FIT-mediated specific phosphorylation of p130Cas and the resulting activation of Rac1 are sufficient to induce membrane ruffling and lamellipodium formation, we observed SYF cells and c-Src reconstituted SYF cells under normal growth conditions.

We quantified membrane ruffling in fixed transfected cells by confocal microscopy, summarized in Table 1, and also monitored living cells by video microscopy. Observation of the membrane dynamics of SYF cells shows a relatively static membrane with few membrane ruffles and lamellipodia (see additional file 1). In contrast, c-Src reconstituted cells showed a dynamic membrane with large membrane ruffles and lamellipodia along with active actin reorganization (see additional file 2), indicating that Src is responsible for these effects. When FIT-compatible Src and p130Cas were transfected in SYF cells, the membrane dynamics approached that of c-Src reconstituted cells, exhibiting extensive membrane ruffling, and lamellipodium formation, and actin reorganization (see additional file 3). Transfection of FIT-compatible Src alone or p130Cas alone in SYF cells did not lead to a significant increase in membrane ruffling or lamellipodium formation (Table 1 and data not shown), indicating that it is indeed the specific phosphorylation of p130Cas that leads to these effects. Quantification of membrane ruffling in fixed cells demonstrates that statistically significant increase in membrane ruffling in SYF cells is observed only when FIT-compatible Src and FIT-compatible p130Cas are co-transfected (Table 1). Thus, we can conclude that the FIT-mediated specific phosphorylation of p130Cas is sufficient to promote increased membrane ruffling and lamellipodia.

**Table 1:** Quantification of membrane ruffling in SYF cells following plasmid transfection

Treatment	Percent cells showing membrane ruffling
Mock	21.9 ± 3.65
ΔSrc	31.4 ± 0.96
ZipA ΔSrc	28.7 ± 2.72
ZipB p130Cas	25.6 ± 1.85
ZipB p130Cas + ΔSrc	32.8 ± 2.14
ZipB p130Cas + ZipA ΔSrc	56.9 ± 0.67*

SYF cells were transfected and fixed as described in Materials and Methods and membrane ruffling observed by confocal microscopy (a membrane ruffle is defined as a narrow, actin-rich upward protrusion of the membrane). Data is expressed as percent mean ± S.E.M. (standard error of mean) of three independent observations and was analyzed by one-way ANOVA (analysis of variance), followed by Tukey's multiple comparison test. * denotes p < 0.001 as compared to ZipA ΔSrc transfected group or ZipB p130Cas transfected group. ΔSrc = v-Src lacking SH2 and SH3 domains, ZipA ΔSrc = ΔSrc containing a coiled-coiled domain (FIT-compatible Src), ZipB p130Cas = p130Cas containing a complimentary coiled-coiled domain (FIT-compatible p130Cas).

The role of p130Cas as an important mediator of cell migration was further corroborated by live cell imaging of MEFs lacking p130Cas, which show similar membrane dynamics as the SYF cells (static membrane with few membrane ruffles or lamellipodia; see additional file 4), while MEFs from wild-type littermates show extensive membrane ruffling, lamellipodium formation and actin reorganization (see additional file 5). Taken together, these results suggest that tyrosine phosphorylation of p130Cas is both necessary and sufficient to induce membrane ruffling in fibroblasts.

### Mechanism of phosphorylation of p130Cas

Since p130Cas can exist in a macromolecular complex with the two NTKs Src and FAK at focal adhesions, a number of studies have been conducted to determine the role of each of these components, i.e., who associates with whom and how, and who phosphorylates whom. A number of different models have been proposed (see [44] for the models and references). Based on methodologies such as in vitro kinase assays [44], antibody labeling [46] or mutational analysis of Src [45] or FAK [16,21], and knockout studies [47,48], it has been proposed that FAK acts as a scaffold to recruit both p130Cas and Src; in this complex, Src phosphorylates p130Cas (and other residues on FAK).

**Figure 6:**
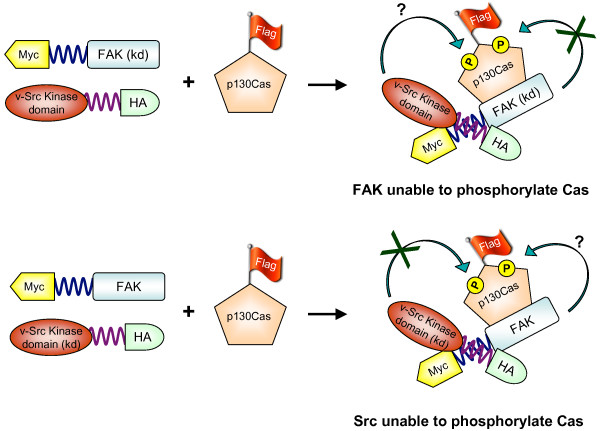
FIT as a tool to elucidate the role of kinases in a multi-molecular complex. FAK, Src and p130Cas are known to exist in multi-molecular complexes. Since both FAK and Src possess kinase activity, either one (or both) could phosphorylate p130Cas. The importance of Src or FAK as the kinase responsible for phosphorylating p130Cas can be easily studied by using kinase-dead (kd) versions of each. From our results, we conclude that the Src kinase activity is responsible for phosphorylating p130Cas.

**Figure 7:**
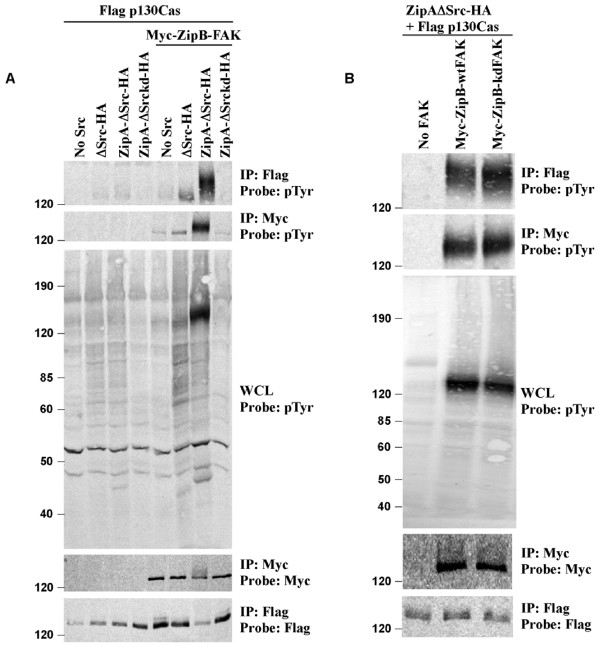
Kinase activity of Src is primarily responsible for phosphorylating p130Cas in focal adhesion complexes with FAK. 293T cells were transfected and subjected to immunoprecipitation and probing as shown. Phosphorylation of p130Cas was absent (A) when Src lacked kinase activity (kd), but not (B) when FAK lacked kinase activity (kd), suggesting that the primary function of FAK is to hold p130Cas and Src in proximity to allow p130Cas phosphorylation by Src. Approximate position of the molecular weight markers is shown on left (in kDa).

In order to obtain direct evidence that it is indeed Src kinase activity and not FAK activity that is responsible for p130Cas phosphorylation, we approached this seemingly complex problem with a very simple experiment utilizing FIT. 293T cells were transfected with Flag-tagged p130Cas, FIT-compatible Myc-tagged FAK, and FIT-compatible HA-tagged Src (Figure 6). Using FIT to force interaction of Src and FAK eliminates any need for kinase activity or phosphorylation to mediate this association. p130Cas was then immunoprecipitated by anti-Flag antibody, and blots probed by anti-pTyr antibody to monitor phosphorylation. As shown in Figure 7, tyrosine phosphorylation of p130Cas was seen only when FAK was present and when Src possessed kinase activity, but not when Src lacked kinase activity (Figure 7A). In contrast, p130Cas was still tyrosine phosphorylated when FAK lacked kinase activity (Figure 7B). Thus we conclude that the primary role of FAK in the complex is to serve as a scaffold to recruit Src and p130Cas to focal adhesions, promoting efficient phosphorylation of p130Cas by Src.

## Discussion

Activated tyrosine kinases associate with and phosphorylate multiple substrates, which can in turn associate with other proteins. This multiplicity of substrates and interactions makes it difficult to determine the exact role of an individual substrate protein in mediating a particular biological process. In practical terms, it is often impossible to study a binding or phosphorylation event in the absence of many other confounding factors. FIT was designed to overcome this limitation by promoting a specific association between two proteins of interest, allowing the biological effects to be assessed. This approach allows upstream activating signals to be bypassed, greatly simplifying experimental analysis by focusing analysis on a single interaction or phosphorylation of a single substrate.

Adaptor and scaffold proteins such as p130Cas are of considerable interest in signal transduction pathways since they can associate with multiple proteins, serving as points for signal convergence and divergence to regulate multiple biological processes. p130Cas can bind to a number of proteins via its SH3 domain, a proline rich region, a substrate domain containing 15 YXXP motifs (consensus sequences for tyrosine phosphorylation, which if phosphorylated can bind to SH2 domain containing proteins), a serine-rich region, and carboxyl terminal domain (see [63] for review and references). p130Cas has been found to be phosphorylated in v-Crk- [38-40] and v-Src- [37] transformed cells. Since p130Cas is expressed ubiquitously and is mainly localized in focal adhesions [18,39,40,58], which regulate biological processes such as cell adhesion, migration, differentiation, proliferation and survival, p130Cas aptly serves as a site of convergence and divergence in cell signaling. For example, mechanical forces (e.g. from tension on actin cables) have been shown to induce a conformational change in p130Cas, leading to its phosphorylation and the activation of downstream signaling [64,65].

Possibly the best evidence that p130Cas plays an important role in cell migration comes from the fact that mice lacking p130Cas were found to die in utero exhibiting several severe developmental defects which were specifically attributed to failure of cell migration [11]. Moreover, fibroblasts derived from these mice had short, thin peripheral actin fibers, as opposed to the long, thick fibers traversing the cell seen in wild type fibroblasts, and exhibited defects in cell migration as well as cell transformation [11,66]. Although the role of p130Cas in cell migration is well documented, it was not known whether phosphorylation of p130Cas is sufficient to initiate cell migration, or if p130Cas is just one of many proteins whose simultaneous phosphorylation is required. In this study, we have used FIT to induce the specific phosphorylation of p130Cas, and show that this is sufficient to initiate downstream signaling and actin reorganization.

SYF cells are an ideal biological system to study FIT-mediated biological responses, as these cells are known to have defects in cell migration and they have a low background level of tyrosine phosphorylation [8,53]. Furthermore, reconstitution of these cells with wt c-Src provides an excellent control for normal Src-initiated signals. This system allows us to induce the phosphorylation of specific substrate proteins by FIT and assess the results on rescue of actin cytoskeletal defects.

Previously, we have shown that FIT-mediated phosphorylation can activate downstream signaling from Stat3 using a luciferase reporter assay [50,51]. However, because it involves elimination of normal upstream signals, it was possible that FIT-mediated phosphorylation of p130Cas might either be insufficient to activate downstream signaling pathways, such as Rac1, or might activate other pathways not normally activated under physiological conditions (if, for example, phosphorylated p130Cas were mislocalized in the cell). In our studies, we were able to detect activated Rac1 after FIT-mediated phosphorylation of p130Cas (Figure 5), thereby suggesting that the downstream signaling pathways normally activated upon phosphorylation of p130Cas signaling are intact. Furthermore, we were unable to detect activation of Erk upon FIT-mediated phosphorylation of p130Cas (data not shown) consistent with other studies showing that Erk activation is independent of phosphorylation of p130Cas [21,24,67]. These results support the premise that FIT is a valuable tool to elucidate the normal physiological responses mediated by the specific phosphorylation of a particular substrate.

Activation of Rac1 due to activation of signaling cascades involving p130Cas phosphorylation is known to increase membrane ruffling and lamellipodial extensions [24,29,30]. The importance of Rac1 in mediating these effects is further corroborated by other studies using dominant negative forms of Rac1, which inhibited membrane ruffling, lamellipodium formation and cell migration [28,31,61,62]. Activation of Rac1 is not the sole determinant of membrane ruffling, however, since Rac1-deficient macrophages exhibit reduced but not absent membrane ruffling [68]. It has been suggested that activation of Rac1 may be necessary primarily for membrane ruffling while activation of Rac2 may be necessary primarily for cell migration (see [68] and references therein).

A recent study has indicated even more complexity to the role of Rac1 in cell migration, indicating that in the absence of chemotactic signals, low levels of activated Rac1 occurred when cells were in a three dimensional matrix promoting a "directionally persistent" cell migration with reduced membrane ruffling and peripheral lamellae, while higher levels of activated Rac1 occurred when cells were in a two dimensional matrix promoting random cell migration with increased membrane ruffling and peripheral lamellae, as seen when cells are exploring their local environments [69]. Furthermore, during chemotaxis, Rac1 may also potentiate the effect of growth factors to stimulate Erk, but Erk activity does not influence Rac1 activity or membrane ruffling [67]. Our live cell microscopy studies (additional files 1, 2, 3, 4, 5) are in agreement with these observations, since FIT-mediated specific phosphorylation of p130Cas (and subsequent activation of Rac1) stimulated the formation of membrane ruffles and lamellipodia (like those exhibited by c-Src reconstituted cells) in SYF cells, which are normally devoid of these structures.

We recently showed that recruitment and subsequent phosphorylation of p130Cas is important for membrane ruffling and actin reorganization following PDGF stimulation of fibroblasts [70]. While that study focused on the role of Nck adaptors in transducing signals from p130Cas, it is entirely consistent with our current data on the importance of p130Cas tyrosine phosphorylation for membrane ruffling and actin reorganization. We show here that FIT-mediated tyrosine phosphorylation of p130Cas is **sufficient **to bring about these effects, clearly demonstrating the usefulness of the FIT approach for dissecting complex biological responses.

The FIT approach also allows us to test experimentally the functional role of individual components of multimolecular complexes, such as the FAK-Src-p130Cas complex found in focal adhesions. Since both FAK and Src possess tyrosine kinase activity, it has been difficult to say with certainty which of the two kinases is responsible for phosphorylating p130Cas. We used a simple FIT experiment to validate the model proposed by Hanks and colleagues [44,46], in which FAK acts as a scaffold while Src is responsible for p130Cas phosphorylation. By allowing us to induce the specific association of Src and FAK, independent of the activity or phosphorylation state of each, FIT allowed us to isolate the importance of Src kinase activity for p130Cas phosphorylation (Figure 7).

In summary, our results strongly suggest that the phosphorylation of p130Cas alone is sufficient to initiate activation of Rac1 and actin reorganization including the formation of membrane ruffles and lamellipodial extensions. Of course, these results do not imply that other substrates of NTKs such as paxillin or cortactin play no role in cell migration; indeed, it is likely that many proteins involved in adhesion and motility are at least partially redundant, and in most cases fibroblasts lacking one or more components are able to adhere and migrate, albeit with decreased efficiency. However the availability of a method to address the sufficiency of a protein phosphorylation for a biological effect provides a valuable complement to knockout and knockdown methods, which can only address whether a protein or its phosphorylation is necessary. Although we cannot exclude the possibility that other substrates that may physically associate with the Src-p130Cas complex, such as FAK or paxillin, may be phosphorylated to some extent in our experiments, anti-pTyr immunoblotting indicates that such phosphorylation, if any, is minor. In any case, we can state with confidence that the association between Src and p130Cas, and the resulting phosphorylation of p130Cas, is the immediate cause of the biological effects that we observe.

## Conclusion

Taken together, we conclude that Src-p130Cas complex formation and the resulting phosphorylation of p130Cas is sufficient to induce events leading to membrane ruffling and Rac activation in fibroblasts. We also conclude that Src is primarily responsible for the tyrosine phosphorylation of p130Cas in complex with FAK at focal adhesions.

## Methods

### Cell lines, plasmids and antibodies

The SYF and c-Src reconstituted SYF MEFs were obtained from ATCC, while MEFs lacking p130Cas (Cas-/-) and wild type littermate control MEFs were obtained from Dr. Amy H. Bouton (University of Virginia School of Medicine). These cells were grown in DMEM supplemented with 10% fetal bovine serum at 37°C and 10% CO_2 _for SYF and c-Src SYF, and 5% CO_2 _for Cas-/- and wild type cells. Expression plasmids with or without leucine zipper segments were made as previously described [50]. Wild-type FAK and K454R (kinase-dead) mutant FAK cDNAs were obtained from Dr. Steven K. Hanks (Vanderbilt University School of Medicine) and were incorporated in pEBB expression plasmids containing an N-terminal Myc tag and Zip B leucine zipper segment. The Rac1 expression plasmid and the Rac1 binding domain of Pak (PBD) in pGEX bacterial expression vector were obtained from Dr. Dianqing Wu (University of Connecticut Health Center, presently at Yale University School of Medicine). The anti-pTyr monoclonal antibody (pTyr 100) was purchased from Cell Signaling Technology, anti-Myc monoclonal (9E10) and polyclonal antibodies and anti-Rac antibody from Santa Cruz Biotechnology, anti-Flag (M2) polyclonal antibody from Sigma and rhodamine-conjugated secondary antibody from Pierce Biotechnology. Monoclonal and polyclonal anti-p130Cas antibodies were obtained from Dr. Amy H. Bouton (University of Virginia School of Medicine). Immunoprecipitation and western blotting studies using antibodies were done as previously described [50].

### Confocal imaging of fixed and live cells

For visualizing fixed cells, a confluent monolayer plate of cells was trypsinized and 1 × 10^5 ^cells were plated on untreated glass coverslips contained in 6-well plates in 2 ml of complete growth medium and incubated overnight. The next day, these cells were transfected with various plasmids along with GFP-actin expression plasmid as a marker, using lipofectamine and plus reagent (Gibco BRL). 24 hours post-transfection, cells were washed with phosphate buffered saline (PBS) and fixed with 4% paraformaldehyde in cytoskeletal buffer [71] containing 1% Triton-X for permeabilization. The coverslips were blocked with 1% donor horse serum and 5% bovine serum albumin for 30 minutes, incubated with pTyr antibody for 45 minutes, washed, incubated with rhodamine conjugated secondary antibody for 45 minutes, washed and mounted on glass slides in Fluoromount-G. The cells were then observed under Zeiss 510 laser scanning confocal microscope.

For visualizing live cells, cells were trypsinized, plated directly in 6-well plates and transfected as outlined above. Six hours post-transfection, cells were trypsinized and a third of these cells were plated onto delta-T dishes (purchased from Bioptechs Inc.) in regular growth medium. The medium was changed to growth medium without phenol red about 30 minutes before imaging. The cells were observed live under Zeiss 510 confocal microscope 24 hours post transfection with a heated objective to maintain the temperature of the medium at 37°C. Five to six Z-slices each about 0.6 μM thick of a single cell were imaged for about 20 minutes and these individual slices were then combined using Imaris software.

### Measurement of Rac1 activity

Rac1 activity was measured as described previously [60]. Briefly, 293T cells were co-transfected with Rac1 cDNA with and other plasmids as indicated. Cell lysates were incubated with GST-PBD (glutathione S-transferase fusion with the Rac binding domain of Pak1) beads, washed, boiled in SDS sample buffer, and separated by SDS-polyacrylamide gel electrophoresis. Activated Rac1 bound to GST-PBD was determined by Western blotting using anti-Rac1 as a probe. Whole cell lysates were also probed with anti-Rac1 to measure the total amount of Rac1 expressed after transfection. Concomitant tyrosine phosphorylation of p130Cas was determined as described previously [50].

## Authors' contributions

AS designed and carried out all the experiments in this study and prepared the manuscript. BJM conceived the FIT methodology, acquired funding, participated in the design and coordination of the study and helped in the final editing of the manuscript. Both the authors have read and approved the final manuscript.

## Supplementary Material

Additional file 1 Live cell microscopy of SYF cells transfected with GFP-actin. Click here for file

Additional file 2 Live cell microscopy of c-Src reconstituted cells transfected with GFP-actin. Click here for file

Additional file 3 Live cell microscopy of SYF cells transfected with GFP-actin, FIT-compatible p130Cas and FIT-compatible v-Src. Click here for file

Additional file 4 Live cell microscopy of p130Cas knockout mouse embryonic fibroblasts transfected with GFP-actin. Click here for file

Additional file 5 Live cell microscopy of wild-type littermate mouse embryonic fibroblasts transfected with GFP-actin. Click here for file
